# Using Automated Machine Learning to Predict the Mortality of Patients With COVID-19: Prediction Model Development Study

**DOI:** 10.2196/23458

**Published:** 2021-02-26

**Authors:** Kenji Ikemura, Eran Bellin, Yukako Yagi, Henny Billett, Mahmoud Saada, Katelyn Simone, Lindsay Stahl, James Szymanski, D Y Goldstein, Morayma Reyes Gil

**Affiliations:** 1 Department of Pathology Albert Einstein College of Medicine Montefiore Medical Center The Bronx, NY United States; 2 Tsubomi Technology The Bronx, NY United States; 3 Department of Epidemiology and Population Health and Medicine Albert Einstein College of Medicine Montefiore Medical Center The Bronx, NY United States; 4 Department of Pathology Memorial Sloan Kettering Cancer Center New York, NY United States; 5 Department of Oncology and Medicine Albert Einstein College of Medicine Montefiore Medical Center The Bronx, NY United States

**Keywords:** automated machine learning, COVID-19, biomarker, ranking, decision support tool, machine learning, decision support, Shapley additive explanation, partial dependence plot, dimensionality reduction

## Abstract

**Background:**

During a pandemic, it is important for clinicians to stratify patients and decide who receives limited medical resources. Machine learning models have been proposed to accurately predict COVID-19 disease severity. Previous studies have typically tested only one machine learning algorithm and limited performance evaluation to area under the curve analysis. To obtain the best results possible, it may be important to test different machine learning algorithms to find the best prediction model.

**Objective:**

In this study, we aimed to use automated machine learning (autoML) to train various machine learning algorithms. We selected the model that best predicted patients’ chances of surviving a SARS-CoV-2 infection. In addition, we identified which variables (ie, vital signs, biomarkers, comorbidities, etc) were the most influential in generating an accurate model.

**Methods:**

Data were retrospectively collected from all patients who tested positive for COVID-19 at our institution between March 1 and July 3, 2020. We collected 48 variables from each patient within 36 hours before or after the index time (ie, real-time polymerase chain reaction positivity). Patients were followed for 30 days or until death. Patients’ data were used to build 20 machine learning models with various algorithms via autoML. The performance of machine learning models was measured by analyzing the area under the precision-recall curve (AUPCR). Subsequently, we established model interpretability via Shapley additive explanation and partial dependence plots to identify and rank variables that drove model predictions. Afterward, we conducted dimensionality reduction to extract the 10 most influential variables. AutoML models were retrained by only using these 10 variables, and the output models were evaluated against the model that used 48 variables.

**Results:**

Data from 4313 patients were used to develop the models. The best model that was generated by using autoML and 48 variables was the stacked ensemble model (AUPRC=0.807). The two best independent models were the gradient boost machine and extreme gradient boost models, which had an AUPRC of 0.803 and 0.793, respectively. The deep learning model (AUPRC=0.73) was substantially inferior to the other models. The 10 most influential variables for generating high-performing models were systolic and diastolic blood pressure, age, pulse oximetry level, blood urea nitrogen level, lactate dehydrogenase level, D-dimer level, troponin level, respiratory rate, and Charlson comorbidity score. After the autoML models were retrained with these 10 variables, the stacked ensemble model still had the best performance (AUPRC=0.791).

**Conclusions:**

We used autoML to develop high-performing models that predicted the survival of patients with COVID-19. In addition, we identified important variables that correlated with mortality. This is proof of concept that autoML is an efficient, effective, and informative method for generating machine learning–based clinical decision support tools.

## Introduction

Many regions worldwide are still fighting the first wave of the COVID-19 pandemic, while other areas that have reopened are experiencing a resurgence of cases [1]. During such an emergent situation, it is important for clinicians to effectively and efficiently triage patients. In recent months, studies have proposed several machine learning models that can accurately predict COVID-19 disease severity. Many of these studies have been successful in generating a high-performing model [2-4]. However, until now, these models have only been trained on one kind of machine learning algorithm, and many researchers have limited the evaluation of their models’ performance to area under the curve (AUC) analysis. Studies have either not reported areas under the precision-recall curve (AUPRCs) or have only reported low AUPRCs. Furthermore, these studies have been difficult to replicate due to hyperparameter tuning. The automation of machine learning end-to-end processes has allowed for the development of simple, fast, and easy-to-replicate models that often outperform manually designed models. This study was designed to (1) optimize the performance of predictive models by using automated machine learning (autoML) to generate various machine learning models and automate hyperparameter optimization; and (2) choose the best performing machine learning model based on AUPRCs.

Artificial intelligence (ie, machine learning) models have often been criticized for being black-box models. We tried to stare into this so-called “black box,” identify the variables that drive model performance, and understand the extent of these variables’ effects on model performance. The interpretability of models is crucial in medical environments; for results to be widely accepted, they must be explainable to medical providers. To assess the correctness of a model, clinicians must be able to use their intuition. Therefore, a model’s response must be understandable to clinicians and comparable to biologically plausible expectations.

In this study, we aimed to generate multiple machine learning models, assess their performance, and select the highest-performing model. After ranking variables by importance, we chose the top 10 most influential variables and retrained the autoML models to generate new models that only used these 10 variables. This was done to create high-performing models with low dimensionality. In addition, we sought to provide interpretable black-box model results to clinicians and patients. Finally, the COVID-19 mortality calculator, which is based on this study, was developed and freely available online as a web application [5].This study provides proof of concept that autoML is an efficient, effective, and informative method for building machine learning–based clinical decision support tools.

## Methods

### Variable Selection and Collection

After conducting a literature review, we selected 48 variables for generating high-performing machine learning models. These variables included demographics such as gender; race; age; comorbidities; physical signs/symptoms; and laboratory test results, such as ferritin, interleukin-6, tumor necrosis factor-α, D-dimer, C-reactive protein, and lactic dehydrogenase (LDH) levels [2-4, 6-12].

Data collection and analysis were approved by the Albert Einstein College of Medicine Institutional Review Board. The data were collected by using Clinical Looking Glass (CLG), which is an interactive software application that was developed at the Montefiore Medical Center. This application is used to evaluate health care quality, effectiveness, and efficiency. CLG integrates clinical and administrative data sets, thereby allowing clinicians to build temporally sophisticated cohorts and assess outcomes [13-16].

We queried the CLG database for patients who were aged >18 years, tested positive for COVID-19 (ie, confirmed with a nasopharyngeal specimen and real-time polymerase chain reaction) within 24 hours before or after admission, and were admitted to our institution from March 1 to July 3, 2020. The index time was when a patient tested positive for COVID-19 based on their real-time polymerase chain reaction results. We investigated a total of 48 variables and used the earliest values that were available within 36 hours before or after the index time. The outcome of interest was mortality from any cause within 30 days after the index time.

### Model Development and Evaluation

We used the open-source H2O.ai autoML package for the R language [17-19]. The package can be downloaded to a local device. This allowed us to avoid uploading patient data to a third-party cloud service. The H2O.ai autoML package trains and cross-validates common machine learning algorithms, such as gradient boosting machine (GBM), extreme gradient boosting (XGBoost), general linear models (GLMs), random forest (RF), and deep learning (DL). In addition, the package trains two types of stacked ensemble models—one based on all previously trained models and another based on the best model of each model family. Additional information on how each model was built and which hyperparameters were tuned via autoML can be found in documentation that is provided by H2O.ai [18,19].

We used the 10-fold cross-validation method to train the autoML model on a randomly selected data set that included 80% of the original data. We then used the trained autoML model to generate 20 models and rank them in order of performance (ie, AUPRC). These 20 models were based on the remaining 20% of the original data set.

The AUPRC is a measure of a model’s predictive performance, which is based on the relationship between the positive predictive value (PPV) for the outcome (ie, death; y-axis) and the model’s sensitivity for detecting patients who actually die (ie, x-axis). For reproducibility, we did not use the DL method and trained each model separately. For convenience, we named the best model that was generated with 48 variables MODEL-48. After creating Shapley additive explanation (SHAP) and partial dependence (PD) plots to evaluate MODEL-48, we selected the 10 most influential variables. We used these 10 variables to repeat model training, model ranking, and the selection of the best performing autoML-generated model. For convenience, we named the best model that was generated with these 10 variables MODEL-10.

To further evaluate MODEL-48 and MODEL-10, we generated a binary classifier (ie, dead or alive within 30 days). We chose a threshold probability that maximized the F2 score of each model. Unlike the F1 score, which gives equal weight to precision (ie, PPV) and sensitivity (ie, recall), the F2 score gives more weight to sensitivity and penalizes a model for generating more false negatives than false positives. As our goal was to identify patients who were at a high risk of death (ie, patients who need more attention and further intervention), our model’s metric of success was based on enhancing its sensitivity for detecting patients who were at risk of death. However, this came with the drawback of overcalling death as a predicted outcome. Sensitivity, specificity, PPVs, and negative predicative values (NPVs) were calculated for each binary classifier. The F-score calculation formula was as follows:

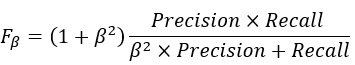



In this formula, to calculate the F2 score, β must equal 2.

### Opening the Black Box: Intuitive Understanding of Model Variable Utility

Once a model determined the most important variable in its internal black box, we used SHAP and PD plots to develop our understanding of the black box. A SHAP plot displays variables in a top-to-bottom format; the most important variable is displayed at the top and the least important variable is displayed at the bottom. Variable importance is determined by the model in question. In this study, SHAP values (ie, x-axis) were indicative of the relative contribution of each patient’s variable values (eg, a systolic blood pressure of 50 mmHg) to the overall prediction of a patient’s mortality. SHAP values of >0 on the x-axis were indicative of variables that contribute to a greater chance of mortality, and SHAP values of <0 were indicative of variables that contribute to a lower chance of mortality. In our SHAP plots, each patient was represented by a dot on a horizontal line (ie, a line for each variable). Each dot’s color reflected patients’ variable values, which were scaled to a normal, color-coded distribution (ie, red indicates large values and blue indicates small values) [20].

A PD plot is a graphical depiction of a variable’s marginal effect on the predicted outcome (ie, mortality). The effect of a variable was measured with mean response values. In this study, mortality had a response value of 1, which indicates a 100% chance of dying. A PD plot can show whether the relationship between a target and a feature is linear, monotonic, or complex [21].

### Choosing the Top 10 Most Important Variables: Dimensionality Reduction

Dimensionality reduction is an important process in machine learning model development. Sometimes, the variables in a model correlate with each other, making them redundant variables (ie, blood urea nitrogen [BUN] level, creatinine level, and estimated glomerular filtration rate are all indicators of renal function). If we could generate a model with a low number of unique variables, we would be able to shorten computation times in real clinical settings. In addition, dimension reduction allows models to overcome data sparsity by using variables that have more data points. Furthermore, by identifying the top 10 most important variables, clinicians can focus on ordering medical tests instead of obtaining data on 48 variables, and machine learning developers can have fewer concerns about handling missing values.

In this study, after evaluating SHAP and PD plots, we chose the 10 most influential variables for generating MODEL-10 (ie, a model that requires only 10 input variables to predict mortality). We first ranked each variable’s influence according to the SHAP values in the highest-performing models. Afterward, we chose variables that were influential in these models. Subsequently, if the rank of a variable was not the same in each model, we chose variables based on clinical insights. Clinically speaking, we wanted at least one unique variable for each biological process (ie, cardiac processes, renal processes, coagulation processes, etc). If there was more than a single variable for describing the same clinical domain or biological process (ie, troponin and probrain natriuretic peptide levels), we chose the variable with the fewest number of missing data points (ie, variables that are commonly ordered by clinicians).

### Handling Missing Values

Different autoML models have different methods for handling missing values. For example, in a tree-based model (ie, GBM, XGBoost, RF models), missing values are interpreted as data that contain information (ie, data that are missing for a reason) instead of data that are missing at random. During tree building, split decisions are made at every node. Each decision is based on the option that minimizes the loss of model functionality and treats missing values as a separate category. This category is then used as the basis for another split decision. Alternatively, GLMs and DL models use the mean imputation method to handle missing values. Further explanations for how each model imputes missing values can be found in documentation that is provided by H2O.ai [18].

**Figure 1:**
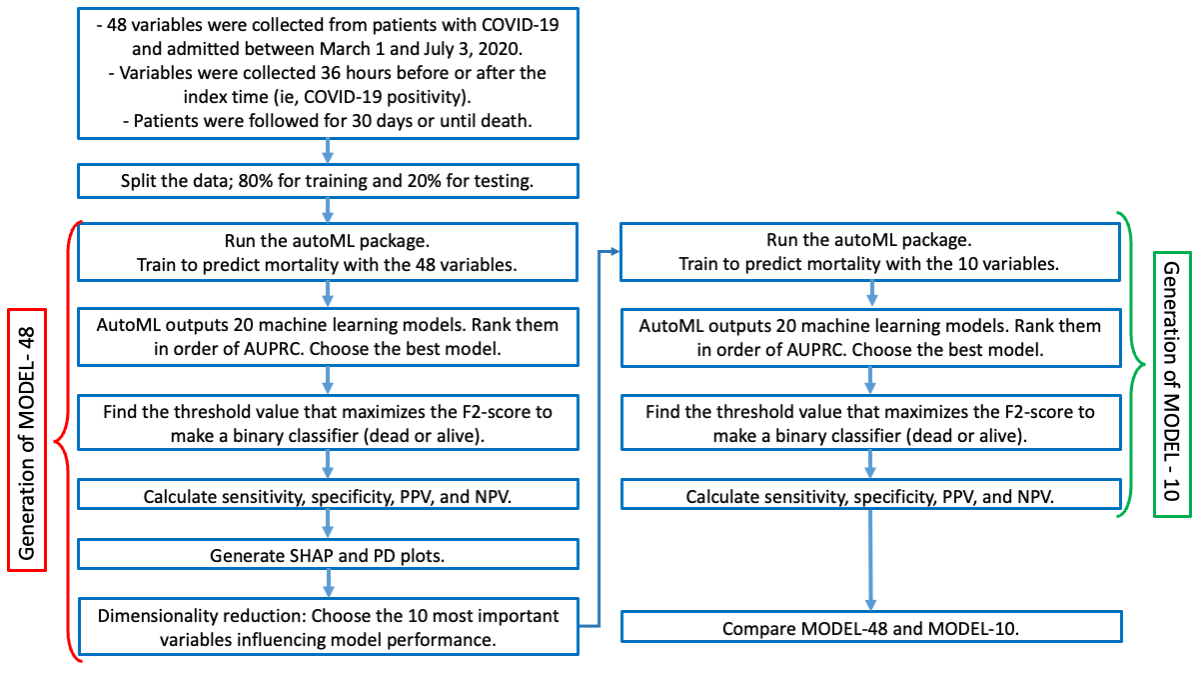
Flowchart summary of our methodology. AutoML: automated machine learning; AUPRC: area under the precision-recall curve; NPV: negative predictive value; PD: partial dependence; PPV: positive predictive value; SHAP: Shapley additive explanation.

The workflow of our study design is depicted in Figure 1.

### Data Access, Responsibility, and Analysis

KI had full access to all the data in this study. KI takes responsibility for the integrity of the data and the accuracy of the data analysis.

## Results

### Study Population

**Figure 2:**
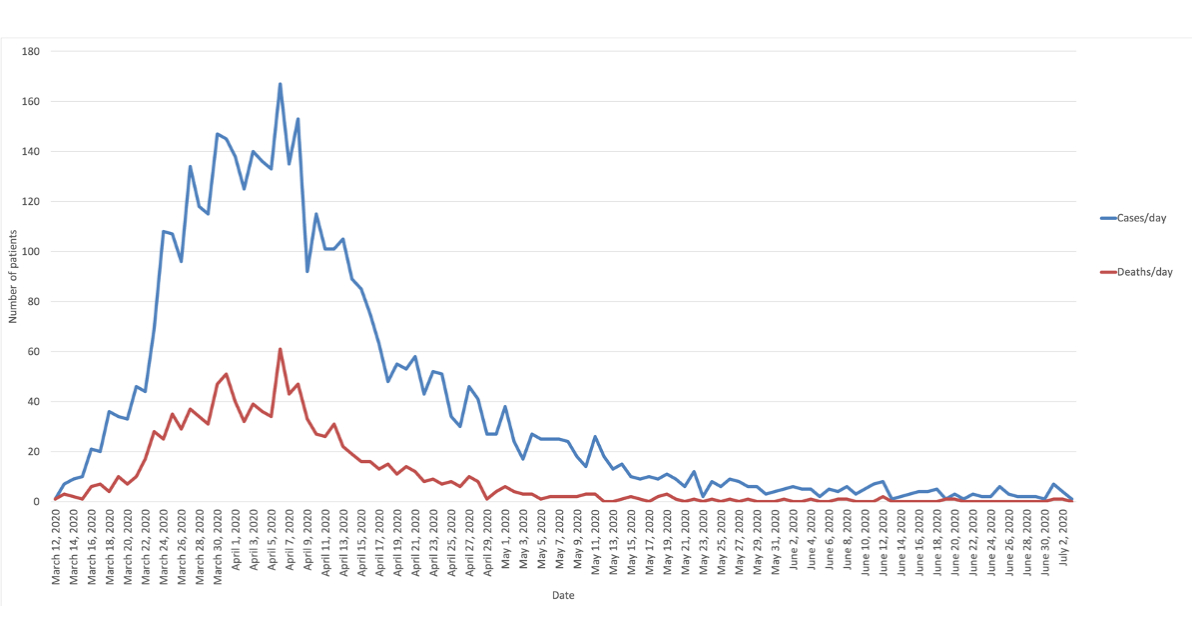
A graph that shows the number of patients who were admitted to the hospital due to SARS-CoV-2 infection (ie, blue line; number of cases per day) and the number of patients who died (ie, red line; number of deaths per day). Data were collected from March 1 to July 3, 2020 at the Montefiore Medical Center.

Between March 1 and July 3, 2020, 4313 adult patients tested positive for COVID-19 and were admitted to a Montefiore Health System hospital within 24 hours of their first COVID-19–positive test. Of these 4313 patients, 1087 (25.2%) died within 30 days of infection (Figure 2).

A summary of case data, patients’ survival rates, and patients’ demographic characteristics is shown in Table 1. The training set consisted of 3468 patients, and the test set consisted of 845 patients. Summaries of the variables for the entire cohort, the training data set, and the testing data sets are provided in Tables S1, S2, and S3 in Multimedia Appendix 1.

**Table 1:** Summary of patients’ demographic characteristics.

Characteristics			Value
Age (years), mean (SD)			63.97 (16.77)
**Gender, n (%)**			
	Male		2289 (53.07)
	Female		2024 (46.93)
Hispanic ethnicity, n (%)			1785 (41.38)
**Race, n (%)**			
	Asian		113 (2.61)
	Black		1560 (36.17)
	White		428 (9.92)
	Other Pacific Islander		4 (0.09)
	Native American or Alaskan		5 (0.12)
	Unknown/undeclared		418 (9.69)
	Other		1785 (41.39)
**Charlson score, mean (SD)**			2.3 (2.34)
	**Charlson score of 1, n (%)**		
		Myocardial infarction	235 (5.44)
		Congestive heart failure	702 (16.28)
		Peripheral vascular disease	145 (3.36)
		Cerebrovascular disease	334 (7.74)
		Dementia	716 (16.6)
		Chronic pulmonary disease	1030 (23.88)
		Rheumatic disease	70 (1.62)
		Peptic ulcer disease	33 (0.77)
		Mild liver disease	197 (4.57)
		Diabetes without chronic complications	616 (14.28)
	**Charlson score of 2, n (%)**		
		Diabetes with chronic complications	999 (23.16)
		Hemiplegia or paraplegia	118 (2.74)
		Renal disease	1314 (30.47)
		Any malignancy	178 (4.13)
	**Charlson score of 3, n (%)**		
		Moderate or severe liver disease	28 (0.65)
	**Charlson score of 6, n (%)**		
		Metastatic solid tumor	58 (1.34)
		AIDS/HIV	40 (0.93)
Survivors after 30 days, n (%)			3226 (74.8)
Length of hospital stay to death (number of days)^a^, mean (SD)			8.4 (6.91)

^a^In total, 1087 patients died within 30 days of infection.

### MODEL-48 Generation and Performance

The output of the 20 machine learning models that were trained via autoML is depicted in Table 2. The best performing model was the stacked ensemble of all machine learning models (AUPRC=0.806). This was MODEL-48. The best performing independent models were the GBM and XGBoost models, which had an AUPRC of 0.803 and 0.793, respectively. The distributed RF model (AUPRC=0.783) came in 14th place and the GLM (AUPRC=0.738) came in last place. The DL model was generated separately from the autoML models for reproducibility purposes. The AUPRC of the DL model plateaued at 0.736.

**Table 2:** Output of the automated machine learning models that used 48 variables. Model ranks are ordered according to AUPRCs^a^.

Rank	Model ID	AUPRC	Area under the curve
1	StackedEnsemble_AllModels_AutoML_20201219_141057	0.807	0.917
2	GBM_2_AutoML_20201219_141057	0.803	0.911
3	StackedEnsemble_BestOfFamily_AutoML_20201219_141057	0.800	0.912
4	XGBoost_grid__1_AutoML_20201219_141057_model_5	0.793	0.907
5	GBM_5_AutoML_20201219_141057	0.792	0.907
6	GBM_3_AutoML_20201219_141057	0.791	0.908
7	XGBoost_2_AutoML_20201219_141057	0.790	0.905
8	XGBoost_grid__1_AutoML_20201219_141057_model_6	0.790	0.910
9	XGBoost_grid__1_AutoML_20201219_141057_model_4	0.788	0.903
10	XGBoost_3_AutoML_20201219_141057	0.788	0.910
11	GBM_grid__1_AutoML_20201219_141057_model_3	0.785	0.909
12	GBM_grid__1_AutoML_20201219_141057_model_2	0.785	0.898
13	GBM_4_AutoML_20201219_141057	0.784	0.914
14	DRF_1_AutoML_20201219_141057	0.784	0.905
15	GBM_grid__1_AutoML_20201219_141057_model_1	0.782	0.913
16	GBM_1_AutoML_20201219_141057	0.781	0.903
17	XGBoost_grid__1_AutoML_20201219_141057_model_1	0.779	0.896
18	XGBoost_grid__1_AutoML_20201219_141057_model_3	0.779	0.909
19	XRT_1_AutoML_20201219_141057	0.775	0.899
20	XGBoost_grid__1_AutoML_20201219_141057_model_2	0.769	0.893
21	XGBoost_1_AutoML_20201219_141057	0.763	0.899
22	GLM_1_AutoML_20201219_141057	0.738	0.877

^a^AUPRC: area under the precision-recall curve.

### Variable Importance

**Figure 3:**
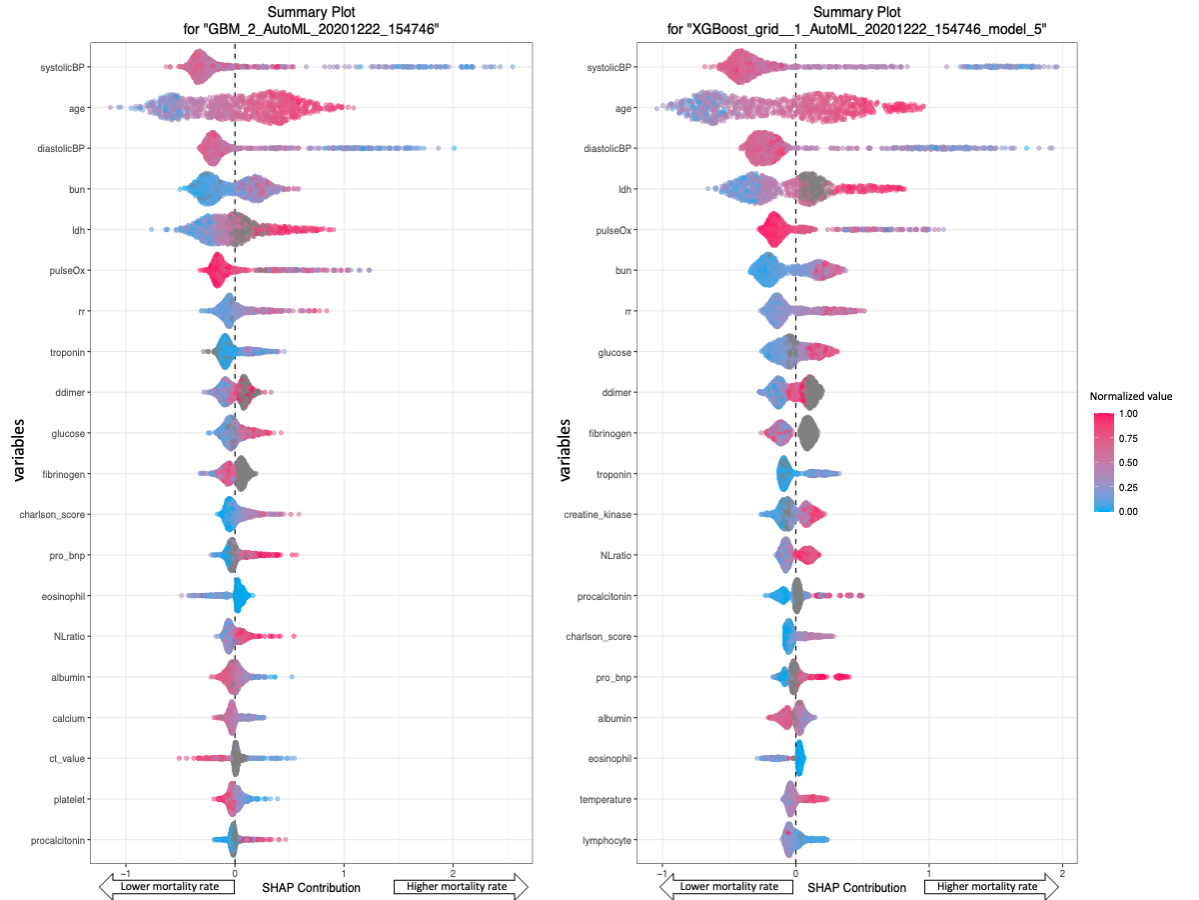
SHAP summary plots of the GBM and XGBoost models. According to the GBM and XGBoost models, higher systolic blood pressure levels (ie, red dots) were associated with a lower probability of death (ie, the left side of the vertical dotted line), and older age (ie, red dots) was associated with higher probability of death (ie, the right side of the vertical dotted line). BUN: blood urea nitrogen; charlson_score: charlson comorbidity index; ct_value: cycle threshold value; diastolicBP: diastolic blood pressure; GBM: gradient boosting machine; LDH: lactate dehydrogenase; NLratio: neutrophil-lymphocyte ratio; pro_bnp: pro-brain natriuretic peptide; pulseOx: pulse oximetry; rr: respiratory rate; SHAP: Shapley additive explanation; systolicBP: systolic blood pressure; XGBoost: extreme gradient boosting.

Figure 3 shows the SHAP plots for the GBM and XGBoost models. In these plots, variables were ranked in descending order of importance. Each patient was represented by one dot on each variable line. The horizontal location of each dot indicated whether the effect of a variable was associated with a higher or lower chance of death [22]. Variable-specific SHAP values of >0 indicated an increased risk of death. For example, the GBM and XGBoost models determined that systolic blood pressure was the most important variable, followed by age and diastolic blood pressure.

**Figure 4:**
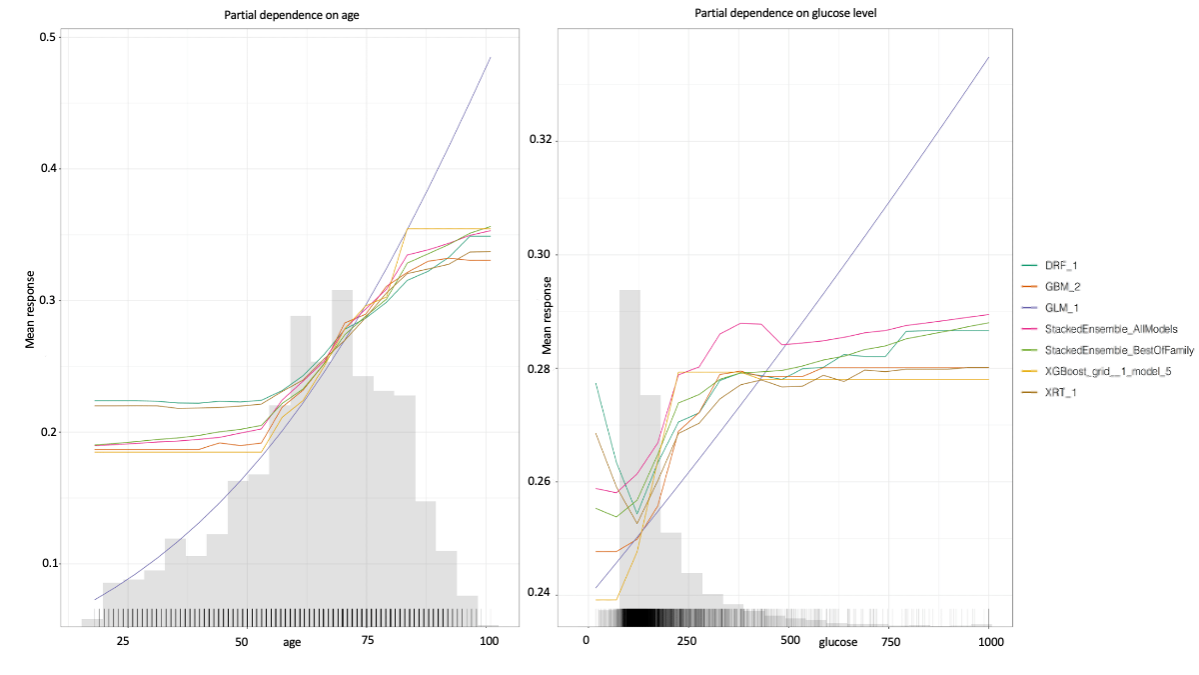
Partial dependence plots for age and glucose level. Partial dependence plots for the other variables are shown in Multimedia Appendix 2. Each line represents a different machine learning algorithm. DRF: distributed random forest; GBM: gradient boosting machine; GLM: generalized linear model; XGBoost, extreme gradient boosting; XRT: extremely randomized trees.

PD plots show the marginal effect that one variable can have on the predicted outcome of a machine learning model. PD plots for the most influential variables are depicted in Multimedia Appendix 2. Each line in a PD plot depicts the best performing model in each machine learning algorithm family. For example, in Figure 4, all models determined that percent mortality increased with age (ie, starting at around 50 years of age). Similarly, all models determined that percent mortality increased with glucose level. However, this was only true for glucose levels of <300 mg/dL (Figure 4).

**Figure 5:**
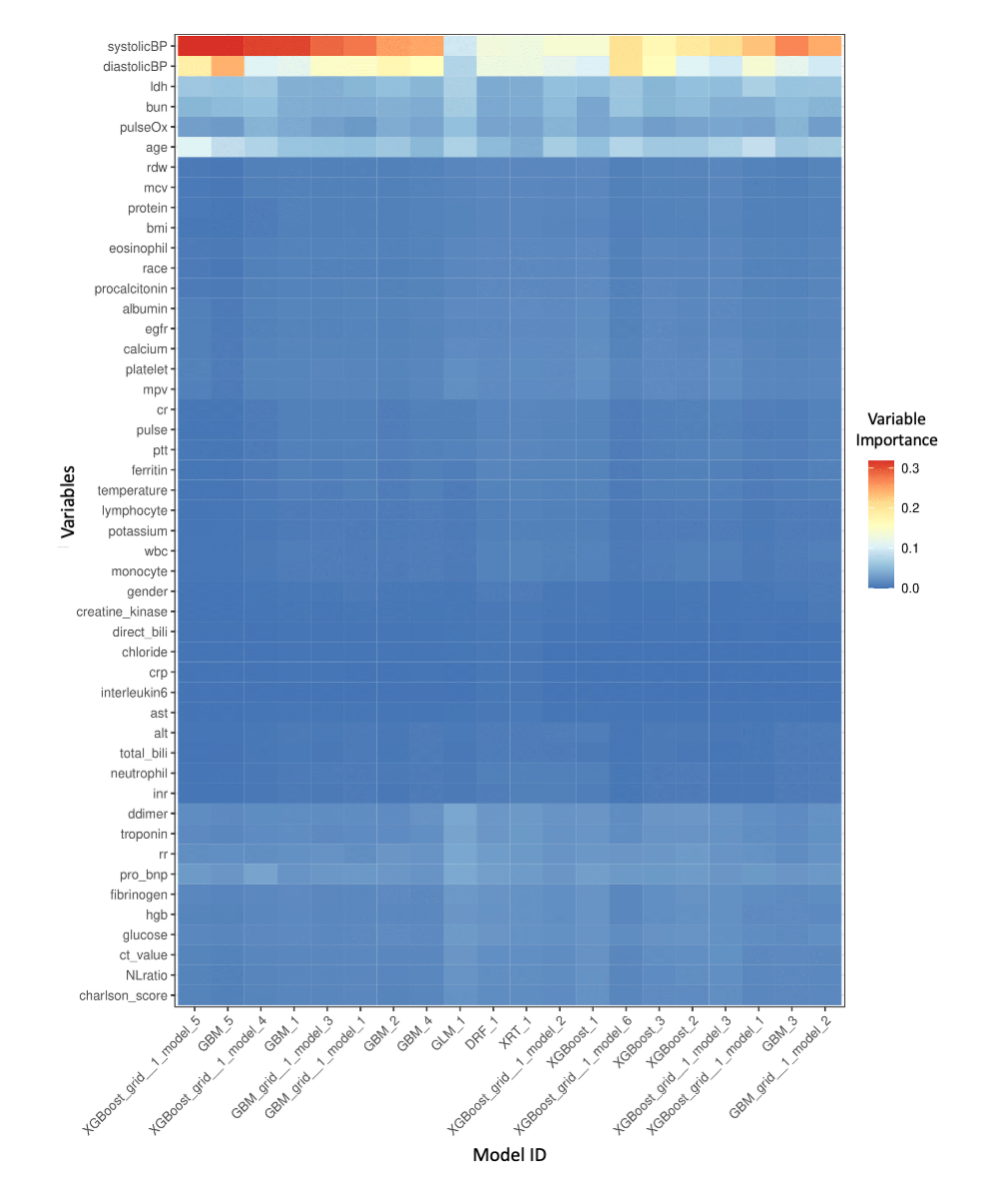
A heatmap that represents the importance of each variable in every machine learning model. DRF: distributed random forest; GBM: gradient boosting machine; GLM: generalized linear model; XGBoost: extreme gradient boosting; XRT: extremely randomized trees.

The importance of each variable in every model is represented on a heatmap in Figure 5.

### Selection of the Top 10 Variables: Dimensionality Reduction

The SHAP plots for the GBM and XGBoost models (ie, the two highest-performing models) showed that systolic and diastolic blood pressure, age, LDH level, pulse oximetry level, respiratory rate, BUN level, and troponin level were top 10 variables in both models. BUN and troponin levels are indicators of renal and cardiac function, respectively. With regard to our marker for coagulation, we chose D-dimer level over fibrinogen level because it ranked higher and had more data points. Furthermore, we used Charlson comorbidity scores to represent comorbidities. Glucose level was also a highly ranked variable. This was likely due to the increased risk of mortality in patients with diabetes. However, since the Charlson comorbidity score also accounts for diabetes, we believed that the Charlson comorbidity score was a more comprehensive predictive variable than glucose level. To confirm whether our choice to use Charlson comorbidity score over glucose level was justified, we trained an autoML model on Charlson comorbidity score data (ie, without glucose level data). We then compared it to a model that was trained on glucose level data (ie, without Charlson comorbidity score data). The former model (AUPRC=0.79) performed better than the latter model (AUPRC=0.78), thereby validating our choice.

According to our variable selection process, the top predictive variables were systolic and diastolic blood pressure, age, LDH level, pulse oximetry level, respiratory rate, BUN level, troponin level, D-dimer level, and Charlson comorbidity score. We believed that these variables provided a good representation of biological processes that are affected by SARS-CoV-2 infection. In addition, these variables are easy to obtain in clinical settings. They also reduce the incidence of missing values.

### MODEL-10 Generation and Performance

We used the top 10 influential variables to generate 20 more machine learning models and rank them in order of AUPRC (Table 3). The best performing model was the stacked ensemble of each machine learning algorithm family (AUPRC=0.791). This was MODEL-10. The best performing independent model was the XGBoost model, which had an AUPRC of 0.790.

**Table 3:** Output of the automated machine learning models that used 10 variables. Model ranks are ordered according to AUPRCs^a^.

Rank	Model ID	AUPRC	Area under the curve
1	StackedEnsemble_BestOfFamily_AutoML_20201219_142406	0.791	0.903
2	XGBoost_grid__1_AutoML_20201219_142406_model_6	0.790	0.894
3	StackedEnsemble_AllModels_AutoML_20201219_142406	0.790	0.903
4	GBM_3_AutoML_20201219_142406	0.782	0.898
5	GBM_5_AutoML_20201219_142406	0.782	0.897
6	DRF_1_AutoML_20201219_142406	0.780	0.899
7	XGBoost_grid__1_AutoML_20201219_142406_model_5	0.777	0.893
8	GBM_2_AutoML_20201219_142406	0.777	0.904
9	XGBoost_grid__1_AutoML_20201219_142406_model_1	0.777	0.896
10	XRT_1_AutoML_20201219_142406	0.776	0.900
11	GBM_grid__1_AutoML_20201219_142406_model_1	0.775	0.899
12	XGBoost_grid__1_AutoML_20201219_142406_model_4	0.775	0.894
13	XGBoost_3_AutoML_20201219_142406	0.772	0.891
14	GBM_grid__1_AutoML_20201219_142406_model_2	0.770	0.896
15	GBM_grid__1_AutoML_20201219_142406_model_3	0.770	0.900
16	GBM_1_AutoML_20201219_142406	0.769	0.895
17	XGBoost_2_AutoML_20201219_142406	0.766	0.890
18	GBM_4_AutoML_20201219_142406	0.766	0.897
19	XGBoost_1_AutoML_20201219_142406	0.762	0.886
20	XGBoost_grid__1_AutoML_20201219_142406_model_3	0.761	0.885
21	XGBoost_grid__1_AutoML_20201219_142406_model_2	0.754	0.889
22	GLM_1_AutoML_20201219_142406	0.733	0.860

^a^AUPRC: area under the precision-recall curve.

### Performance of MODEL-48 and MODEL-10 as Binary Classifiers

**Figure 6:**
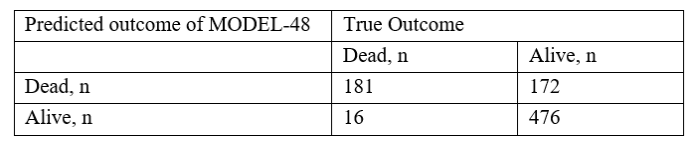
Binary classifier of MODEL-48. The model had an optimized F2-score threshold. This classifier had a sensitivity of 0.92, a specificity of 0.74, a positive predictive value of 0.51, and a negative predictive value of 0.97.

**Figure 7:**
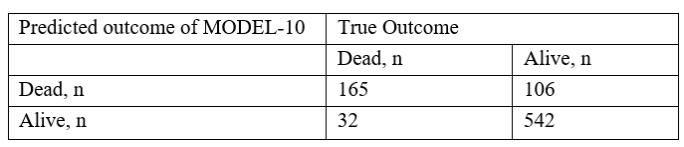
Binary classifier of MODEL-10. The model had an optimized F2-score threshold. This classifier had a sensitivity of 0.84, a specificity of 0.84, a positive predictive value of 0.61, and a negative predictive value of 0.94.

The maximum F2 score of MODEL-48 was 0.793, and the probability threshold was 0.110. The binary classifier for this threshold had a sensitivity, specificity, PPV, and NPV of 0.919, 0.735, 0.513, and 0.968, respectively (Figure 6). The maximum F2 score of MODEL-10 was 0.779, and the probability threshold was 0.202. The binary classifier for this threshold had a sensitivity, specificity, PPV, and NPV of 0.838, 0.836, 0.609, and 0.944, respectively (Figure 7).

## Discussion

### Principal Findings

We were able to use autoML and clinical values (ie, those that were collected early during a patient’s admission to a hospital) to successfully generate multiple machine learning models, assess their performance, and select the highest-performing models for predicting patients’ chances of surviving a SARS-CoV-2 infection. In addition, our study demonstrates that machine learning models that only use 10 clinical variables can predict survival. These models also had high sensitivity values, specificity values, and NPVs. Therefore, autoML is an efficient, informative, and easily reproducible method. The clinical implementation of autoML-based models may require further investigation. However, we demonstrate that autoML is a comprehensive approach for building machine learning–based clinical decision support tools.

Our results show that the best models were GBM and XGBoost models. They both had high performance, as determined by their AUPRCs and AUCs. The RF model, DL model, and GLM performed substantially worse compared to the GBM and XGBoost models. The DL model may have performed better if we had a larger data set, but our DL model required much longer training times than the other models. Tree-based machine learning algorithms (eg, GBM, XGBoost, and RF) are more efficient, and possibly more effective, than neural network algorithms in terms of analyzing tabular data. We used the AUPRC as our metric of model utility because it accounts for the two critical clinical performance metrics that were of specific interest to us—the positive predictive value and sensitivity. We wanted to identify patients who were likely to die so that we could take action and treat as many patients as possible. Alternatively, the AUC accounts for model sensitivity and specificity and ignores the effects of mortality prevalence on model performance. The prevalence of mortality sets the context in which the model must perform; without this information, the model is irrelevant.

Machine learning models can be used to enhance electronic medical record systems and calculate the values of variables that are collected from patients. Based on the performance of MODEL-10, our dimensionality reduction process was successful; 10 variables were enough to generate high-performing models. This shows that not all parameters are necessary for performing calculations and making predictions. Clinicians and hospitals should begin the patient assessment process by prioritizing the ordering of medical tests (ie, tests for the 10 variables). Dimensionality reduction not only reduced the number of variables we needed to consider, but also minimized the number of missing values in the data set and reduced the risk of imputation bias. This may be the reason why the performance of MODEL-10 was similar to that of MODEL-48. These 10 variables may also help researchers with conducting studies on unique cohorts and reproducing our results.

The purpose of autoML is not limited to predicting the survival of patients with COVID-19. AutoML can be used to generate models that are based on other types of clinical data and predict other outcomes (eg, models for predicting which patients require a ventilator). We hope that our study helps other researchers with applying our autoML approach, accelerating the implementation of artificial intelligence models into medical systems, and delivering better medical care.

### Clinical Insights From the Black Box

The trade-off between predictive power and interpretability is a common issue when working with black-box models, especially in medical environments where results have to be explained to medical providers and patients. Interpretability is crucial for questioning, understanding, and trusting artificial intelligence and machine learning systems. According to our variable importance heatmap (Figure 5), many models determined that age, pulse oximetry level, and systolic and diastolic blood pressure were important variables for predicting the outcome. Biomarkers such as BUN level, LDH level, estimated glomerular filtration rate, and probrain natriuretic peptide level were also influential variables. These findings show that our model results are in line with the clinical findings of other researchers [23-27].

Our SHAP and PD plots provided insight into the black box. The SHAP plots allowed us to determine the importance of variables and provided information on how the variables influenced models’ predictions. Alternatively, PD plots provide numerical information on a variable’s effects. For example, the SHAP plots for the GBM and XGBoost models showed that high glucose levels were associated with an increased probability of mortality (ie, high SHAP value). Additionally, the PD plots showed that increases in glucose level were proportional to increases in patients’ chances of death. However, this was only true for glucose levels of <300 mg/dL. These results may support the idea that people with diabetes are at an increased risk of mortality. Therefore, SHAP and PD plots can be used to confirm clinical findings and show clinical thresholds.

Other variables that were less influential are also worthy of examination. For example, the SHAP plots (Figure 3) for the GBM model showed that low albumin levels were weakly associated with an increased chance of death. Such findings may provide insight into the disease mechanism of COVID-19. In addition, models that only use 10 variables can be implemented by institutions that might not be able to collect all 48 variables that were tested in this study. If high-performing models can be generated with only 10 variables, clinicians and hospitals can focus on collecting these variables when conducting patient assessments. Such models also minimize the problem of data sparsity and the risk of imputing missing data points. In addition, the use of such models will help clinicians with manually entering values (eg, inputting values on a mobile device).

### Limitations and Future Work

We recognize that there were limitations to our study. Our cohort was limited to patients with severe conditions that required them to be admitted to a hospital. Therefore, our findings may not be generalizable to all patients with COVID-19. For example, we were surprised to learn that our machine learning models did not identify race as an important predictor of death, given the fact that at a population level, the relative risk of mortality among Black patients is higher than that of White patients [28]. However, one population study analyzed the relative risk of mortality among Black patients and White patients with COVID-19 who were admitted to our institution (ie, Montefiore Medical Center). This study found that there were no considerable differences in mortality rates between the two groups once patients were admitted to our hospital [28]. During a pandemic, hospital beds are scarce. Therefore, only patients who exhibit severe symptoms are allowed to be admitted to emergency rooms. Fortunately, our colleagues from our institution conducted a conventional logistic regression analysis, which resulted in the same finding; hospitalized White and Black patients with illnesses of equal severity and relevant predictor’s for disease progression at admission had similar mortality rates [28]. The results from our machine learning models are in line with those of the logistic regression analysis.

In our study, systolic and diastolic blood pressure were the most important variables. However, these variables may simply indicate that patients with severe illnesses and hypotension are at an imminent risk of death. Temporal features were not considered in our analysis. For example, we did not determine whether hypotension at admission was an important variable for patients who survive during the first 24 hours of admission. Further, we did not determine whether a variable’s importance diminishes in populations that survive after the first 48 or 72 hours of admission. In a future study, we would like to test whether our models are robust enough to predict death during different times of admission. For example, we would test our models’ performance for predicting the death of patients within the first week of admission and the fourth week after admission.

The handling of missing values is a challenging problem in machine learning model development. Fibrinogen, procalcitonin, and cycle threshold values were missing for many people in our cohort (Table S1 in Multimedia Appendix 1). We understand that missing values are not indicative of a variable’s clinical importance. For example, changes in practice patterns alter the meaning of missing data. After the first few weeks of the COVID-19 pandemic, our institution implemented a best practice protocol that required clinicians to measure patients’ D-dimer levels upon admission and recommend anticoagulation treatment to patients with elevated D-dimer levels. Clearly, the presence of D-dimers during the early period of the pandemic had a different meaning from that of the presence of D-dimers during the later period of the pandemic. Similarly, missing D-dimer level values were considered random events during the later period of the pandemic and purposeful events during the early period of the pandemic [29]. Therefore, missing data from the early period of the pandemic have a different meaning compared to that of missing data from the later period. However, our imputation methods did not account for temporal changes in the meaning of missing data. This is an important challenge that must be considered in future machine learning software development studies.

With regard to the generalization of our model, future studies need to be conducted to assess whether our models are useful at other institutions during the second wave of COVID-19. As patient demographics can differ from institution to institution, hospitals may need to customize their models in accordance with their patient populations. Models should also be designed to integrate new data and adjust to the ever-changing environment. We are continually working on reinforcement learning methods for updating our model in real time.

### Conclusion

We used autoML to generate high-performing machine learning models that predicted the mortality of patients with COVID-19. We also identified important variables that were strongly associated with patients’ survival. Our study provides proof of concept that autoML is an efficient, effective, and informative method for training machine learning models and gaining insight into disease processes. AutoML models may help clinicians with triaging patients during the COVID-19 pandemic. Our COVID-19 mortality calculator, which is based on this study, is freely available online as a web-based computer application [5].

## Appendix

Multimedia Appendix 1 Summary of all variables that were collected from the entire cohort and used in the training and testing data sets.

Multimedia Appendix 2 Partial dependence plots of the most important variables.

## Abbreviations

AUC: area under the curve
AUPRC: area under the precision-recall curve
autoML: automated machine learning
BUN: blood urea nitrogen
CLG: Clinical Looking Glass
DL: deep learning
GLM: general linear model
GBM: gradient boosting machine
LDH: lactate dehydrogenase
NPV: negative predictive value
PD: partial dependence
PPV: positive predictive value
RF: random forest
SHAP: Shapley additive explanation
XGBoost: extreme gradient boosting
