# Supplementary material for: Using Automated Machine Learning to Predict the Mortality of Patients With COVID-19: Prediction Model Development Study
Source: J Med Internet Res. 2021 Feb 26;23(2):e23458. doi: 10.2196/23458 (PMC7919846; doi:10.2196/23458)
Supplement: Multimedia Appendix 1 [file jmir_v23i2e23458_app1.pdf]

Table S1: Summary of variables from the entire cohort.

variable no.	variables	n	% missing	mean	standard deviation
1	Age	4313	0.00	64.00	16.80
2	Gender	4313	0.00	1.53	0.50
3	Race	4313	0.00	3.81	1.75
4	Albumin	4009	7.05	3.72	0.54
5	Diastolic BP	4313	0.00	67.60	16.60
6	Systolic BP	4313	0.00	120.00	24.70
7	Cr	4134	4.15	2.04	2.59
8	D-Dimer	2746	36.33	3.97	5.52
9	eGRF	4133	4.17	63.30	35.70
10	Eosinophil	4265	1.11	0.04	0.10
11	Ferritin	2405	44.24	1360.00	2960.00
12	Fibrinogen	1991	53.84	621.00	202.00
13	Hgb	4265	1.11	12.60	2.32
14	INR	3770	12.59	1.23	0.95
15	Lymphocyte	4265	1.11	1.35	4.98
16	Neutrophil	4265	1.11	6.74	4.17
17	N/L Ratio	4265	1.11	7.87	8.76
18	Platelet	4265	1.11	234.00	108.00
19	Protein	3994	7.40	7.08	0.77
20	Pulse	4279	0.79	98.90	20.50
21	Pulse Ox	4277	0.83	92.70	8.36
22	RR	4279	0.79	21.20	5.82
23	Temperature	4276	0.86	99.20	1.68
24	WBC	4265	1.11	8.85	7.25
25	ALT	4004	7.16	44.50	115.00
26	AST	4093	5.10	0.30	0.56
27	BUN	4134	4.15	32.70	32.60
28	Calcium	4240	1.69	8.83	0.75
29	Chloride	4093	5.10	0.30	0.56
30	CRP	4093	5.10	0.30	0.56
31	Interleukin 6	4093	5.10	0.30	0.56
32	LDH	3212	25.53	455.00	359.00
33	MCV	4265	1.11	89.40	7.37
34	Monocyte	4265	1.11	0.61	1.10
35	MPV	4161	3.52	11.00	1.11
36	Procalcitonin	2113	51.01	2.37	7.63
37	RDW	4263	1.16	14.50	2.15
38	Troponin	3601	16.51	0.06	0.26
39	PTT	3359	22.12	35.40	14.70
40	BMI	4070	5.63	30.40	48.40
41	Glucose	3711	13.96	188.00	133.00
42	Direct Bilirubin	4093	5.10	0.30	0.56
43	Total Bilirubin	4093	5.10	0.60	0.94
44	Creatine Kinase	3342	22.51	642.00	3110.00
45	Pro BNP	2267	47.44	2410.00	4330.00
46	Potassium	4158	3.59	4.42	0.74
47	Charlson Score[a]	4313	0.00	2.30	2.34
48	Ct_value[b]	1088	74.77	27.70	6.17
Survival outcome		4313			
	Survived	3226			
	Died	1087			

Abbreviations: systolicBP, systolic blood pressure; diastolicBP, diastolic blood pressure; cr, creatinine; egfr, estimated glomerular filtration rate; hgb, haemoglobin; inr, international normalized ratio; NLratio, neutrophil-lymphocyte ratio; rr, respiratory rate; wbc, white blood cell count; alt, alanine transaminase; ast, aspartate transaminase; bun, blood urea nitrogen; crp, C-reactive protein; ldh, lactate dehydrogenase; mcv, mean corpuscular volume; mpv, mean platelet volume; rdw, red blood cell distribution width; ptt, partial thromboplastin time; bmi, body mass index; direct_bili, direct bilirubin; total_bili, total bilirubin; pro_bnp, pro-brain natriuretic peptide; charlson_score, charlson comorbidity index; ct_value, cycle threshold value.

a. Comorbidities were compiled as Charleston comorbidity score without age.

b. We obtained the cycle threshold values (Ct-values) from positive nasopharyngeal specimens collected in accordance with CDC guidance. Real-time RT-PCR was performed using the Hologic Panther Fusion SARS-CoV-2 assay.

Table S2: Summary of variables from the training dataset.

variable no.	variables	n	% missing	mean	standard deviation
1	Age	3468	0.00	64.00	16.80
2	Gender	3468	0.00	1.53	0.50
3	Race	3468	0.00	3.80	1.74
4	Albumin	3215	7.05	3.72	0.54
5	Diastolic BP	3468	0.00	67.50	16.50
6	Systolic BP	3468	0.00	120.00	24.70
7	Cr	3316	4.15	2.04	2.57
8	D-Dimer	2209	36.33	3.83	5.41
9	eGRF	3315	4.17	63.20	35.80
10	Eosinophil	3431	1.11	0.04	0.10
11	Ferritin	1933	44.24	1360.00	3090.00
12	Fibrinogen	1604	53.84	615.00	199.00
13	Hgb	3431	1.11	12.60	2.30
14	INR	3023	12.59	1.23	0.98
15	Lymphocyte	3431	1.11	1.32	4.24
16	Neutrophil	3431	1.11	6.71	4.10
17	N/L Ratio	3431	1.11	7.83	8.09
18	Platelet	3431	1.11	233.00	108.00
19	Protein	3203	7.40	7.07	0.77
20	Pulse	3441	0.79	98.70	20.60
21	Pulse Ox	3439	0.83	92.70	8.40
22	RR	3441	0.79	21.20	5.80
23	Temperature	3438	0.86	99.20	1.69
24	WBC	3431	1.11	8.80	6.77
25	ALT	3211	7.16	44.30	122.00
26	AST	3292	5.10	0.30	0.60
27	BUN	3316	4.15	32.80	32.90
28	Calcium	3410	1.69	8.83	0.75
29	Chloride	3292	5.10	0.30	0.60
30	CRP	3292	5.10	0.30	0.60
31	Interleukin 6	3292	5.10	0.30	0.60
32	LDH	2591	25.53	456.00	380.00
33	MCV	3431	1.11	89.30	7.27
34	Monocyte	3431	1.11	0.62	1.22
35	MPV	3344	3.52	11.00	1.10
36	Procalcitonin	1705	51.01	2.35	7.50
37	RDW	3429	1.16	14.50	2.17
38	Troponin	2887	16.51	0.06	0.29
39	PTT	2692	22.12	35.30	13.80
40	BMI	3276	5.63	30.50	53.80
41	Glucose	2983	13.96	188.00	132.00
42	Direct Bilirubin	3292	5.10	0.30	0.60
43	Total Bilirubin	3292	5.10	0.61	1.02
44	Creatine Kinase	2683	22.51	649.00	3080.00
45	Pro BNP	1848	47.44	2470.00	4380.00
46	Potassium	3351	3.59	4.43	0.75
47	Charlson Score[a]	3468	0.00	2.33	2.37
48	Ct value[b]	870	74.77	27.50	6.17
Survival outcome		3468			
	Survived	2578			
	Died	890			

Table S3: Summary of variables from the testing dataset.

variable no.	variables	n	% missing	mean	standard deviation
1	Age	845	0.00	63.60	16.70
2	Gender	845	0.00	1.53	0.50
3	Race	845	0.00	3.34	1.33
4	Albumin	794	6.04	3.72	0.54
5	Diastolic BP	845	0.00	68.10	16.90
6	Systolic BP	845	0.00	120.00	24.50
7	Cr	818	3.20	2.03	2.65
8	D-Dimer	537	36.45	4.54	5.92
9	eGRF	818	3.20	63.50	35.20
10	Eosinophil	834	1.30	0.04	0.09
11	Ferritin	472	44.14	1340.00	2340.00
12	Fibrinogen	387	54.20	645.00	212.00
13	Hgb	834	1.30	12.70	2.39
14	INR	747	11.60	1.23	0.87
15	Lymphocyte	834	1.30	1.44	7.25
16	Neutrophil	834	1.30	6.86	4.47
17	N/L Ratio	834	1.30	8.06	11.10
18	Platelet	834	1.30	238.00	112.00
19	Protein	791	6.39	7.10	0.77
20	Pulse	838	0.83	99.80	20.30
21	Pulse Ox	838	0.83	92.50	8.20
22	RR	838	0.83	21.00	5.89
23	Temperature	838	0.83	99.20	1.64
24	WBC	834	1.30	9.04	8.95
25	ALT	793	6.15	45.60	82.60
26	AST	801	5.21	0.28	0.32
27	BUN	818	3.20	32.40	31.60
28	Calcium	830	1.78	8.85	0.76
29	Chloride	801	5.21	0.28	0.32
30	CRP	801	5.21	0.28	0.32
31	Interleukin 6	801	5.21	0.28	0.32
32	LDH	621	26.51	450.00	252.00
33	MCV	834	1.30	89.50	7.77
34	Monocyte	834	1.30	0.59	0.36
35	MPV	817	3.31	11.00	1.13
36	Procalcitonin	408	51.72	2.49	8.20
37	RDW	834	1.30	14.50	2.09
38	Troponin	714	15.50	0.05	0.10
39	PTT	667	21.07	35.80	18.00
40	BMI	794	6.04	29.90	7.38
41	Glucose	728	13.85	188.00	140.00
42	Direct Bilirubin	801	5.21	0.28	0.32
43	Total Bilirubin	801	5.21	0.57	0.50
44	Creatine Kinase	659	22.01	615.00	3230.00
45	Pro BNP	419	50.41	2140.00	4110.00
46	Potassium	807	4.50	4.42	0.69
47	Charlson Score[a]	845	0.00	2.18	2.18
48	Ct value[b]	218	74.20	28.40	6.13
Survival outcome		845			
	Survived	648			
	Died	197			
