# Supplementary figures and images for: Using Automated Machine Learning to Predict the Mortality of Patients With COVID-19: Prediction Model Development Study
Source: J Med Internet Res. 2021 Feb 26;23(2):e23458. doi: 10.2196/23458 (PMC7919846; doi:10.2196/23458)

Partial dependence plot: Mean response (mortality) of 1 will mean 100% chance of dying.

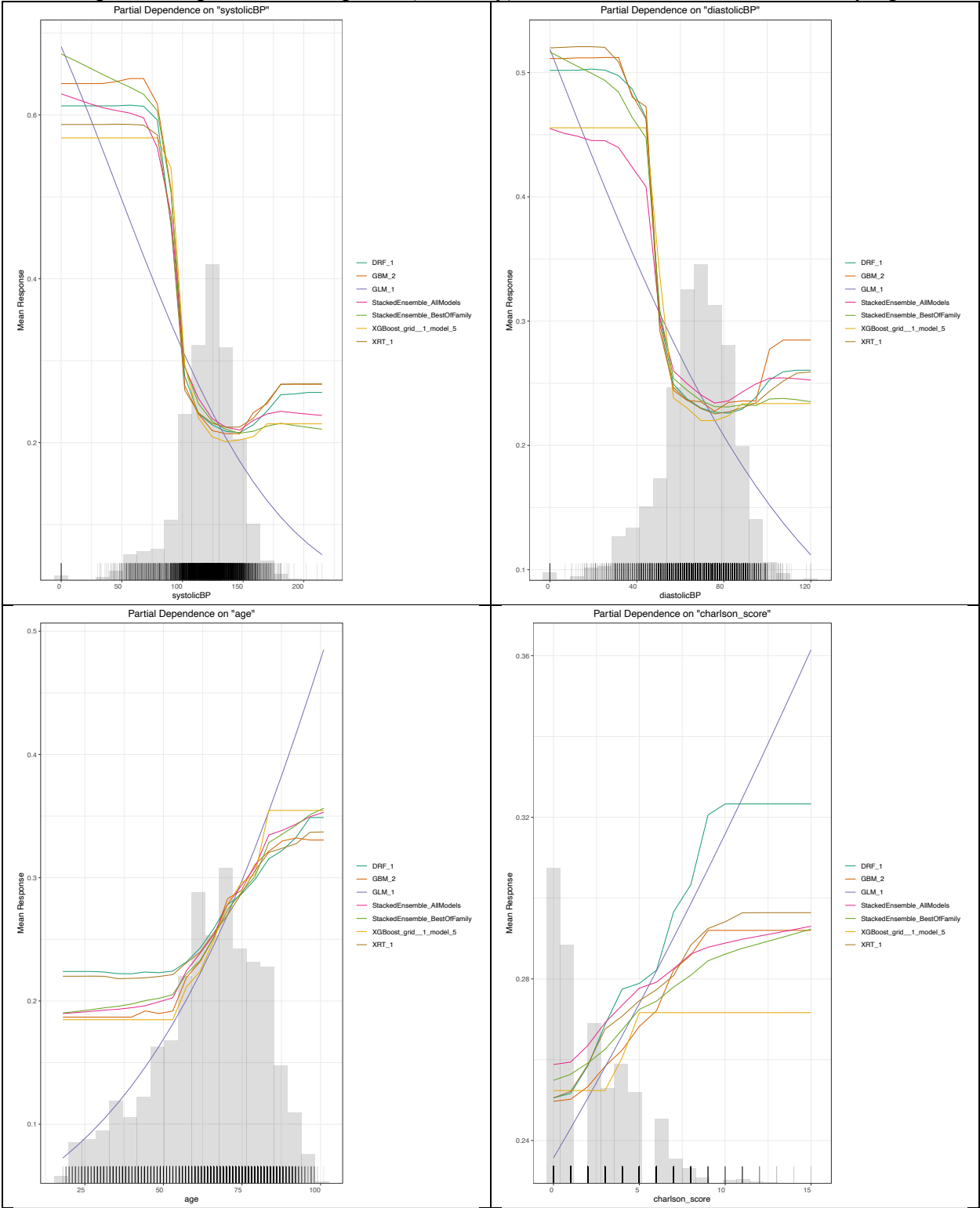

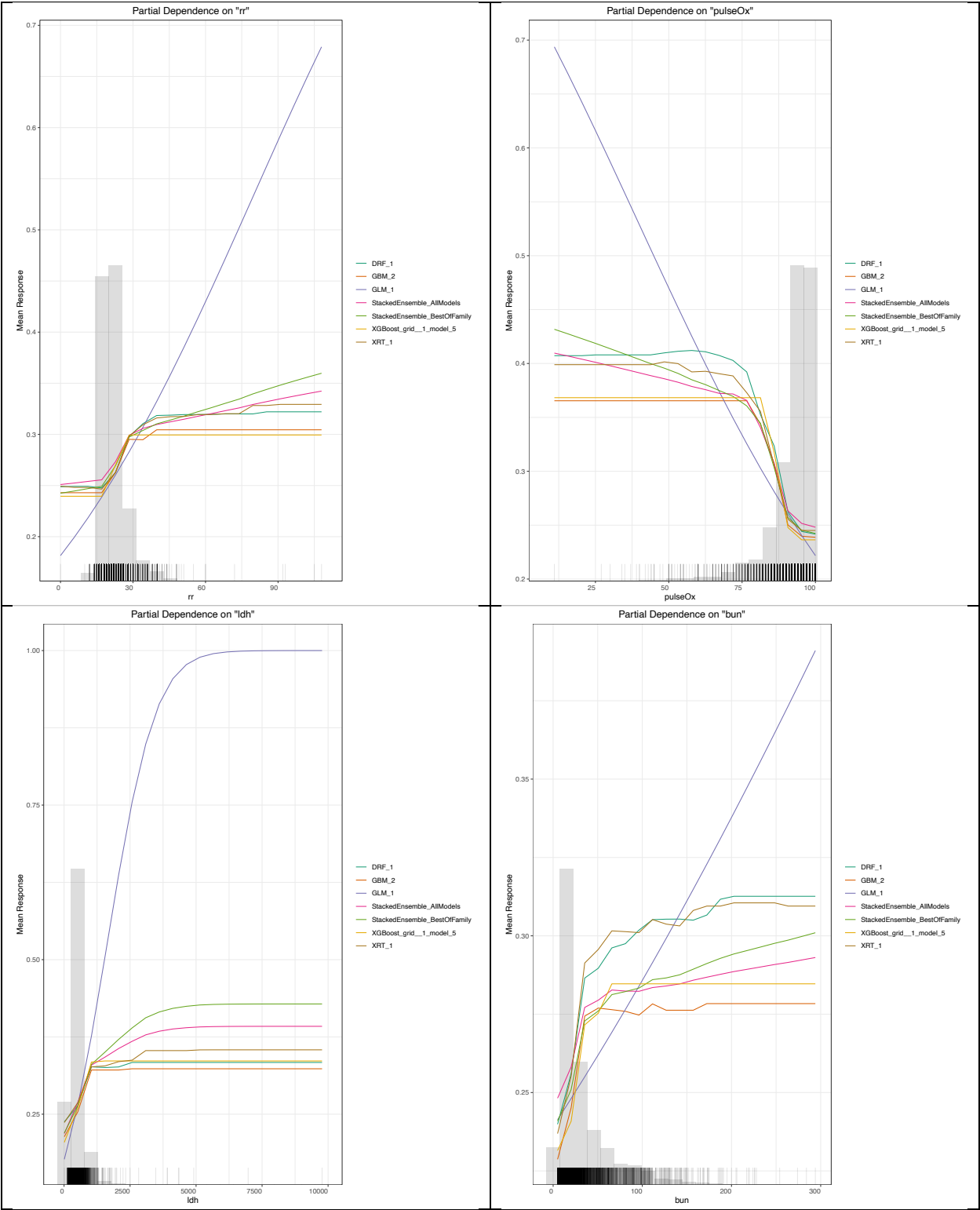

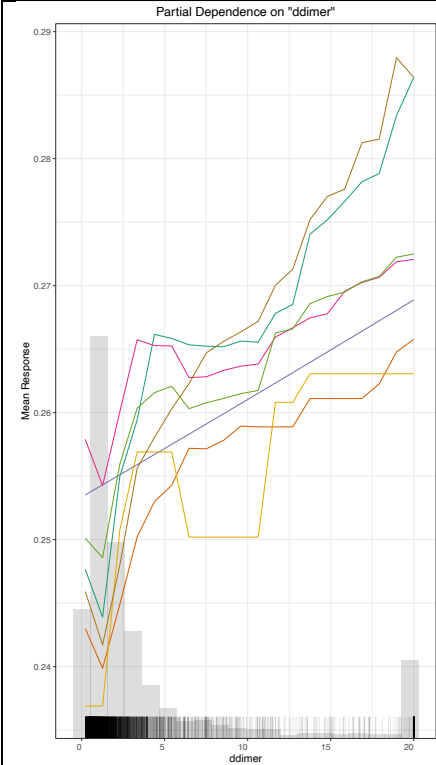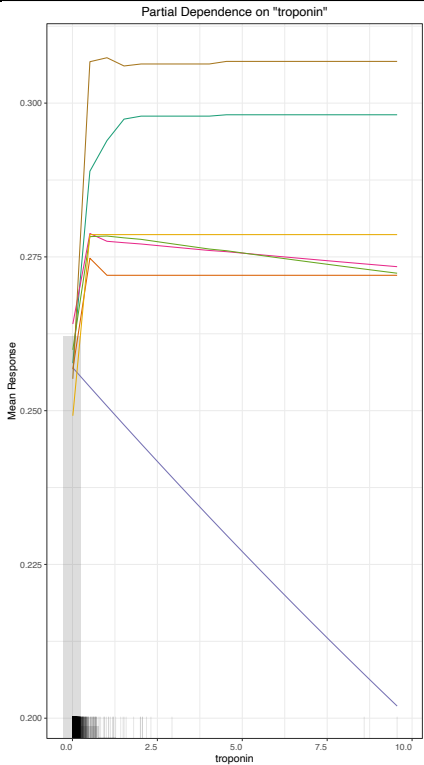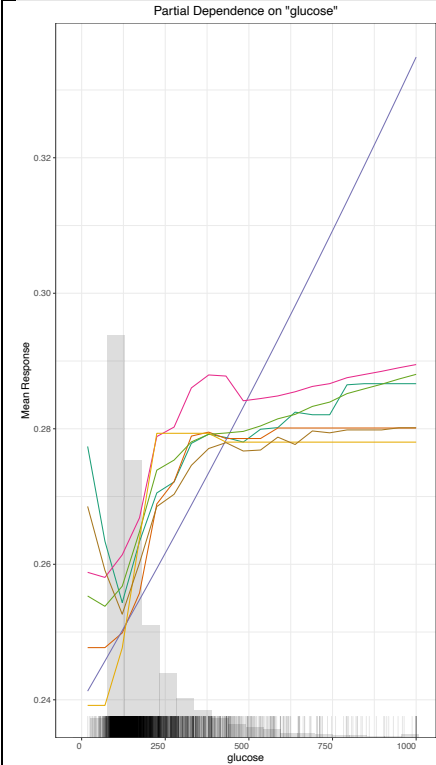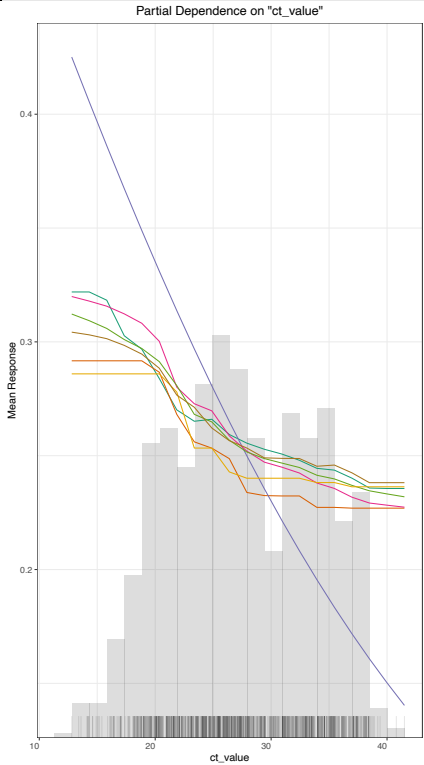

Supplement: Multimedia Appendix 2 [file jmir_v23i2e23458_app2.pdf]
